# Exploring the Biotechnological Value of Marine Invertebrates: A Closer Look at the Biochemical and Antioxidant Properties of *Sabella spallanzanii* and *Microcosmus squamiger*

**DOI:** 10.3390/ani11123557

**Published:** 2021-12-14

**Authors:** Yu-Lun Pan, Maria João Rodrigues, Catarina G. Pereira, Sofia Engrola, Rita Colen, Inês Mansinhos, Anabela Romano, Paula B. Andrade, Fátima Fernandes, Luísa Custódio

**Affiliations:** 1 Centre of Marine Sciences (CCMAR), Faculty of Sciences and Technology, Campus of Gambelas, Ed. 7, University of Algarve, 8005-139 Faro, Portugal; yulun.pan@imbrsea.eu (Y.-L.P.); mjrodrigues@ualg.pt (M.J.R.); cagpereira@ualg.pt (C.G.P.); sengrola@ualg.pt (S.E.); rcolen@ualg.pt (R.C.); 2 MED–Mediterranean Institute for Agriculture, Environment and Development, Faculty of Sciences and Technology, Campus de Gambelas, Ed. 8, University of Algarve, 8005-139 Faro, Portugal; ifmansinhos@ualg.pt (I.M.); aromano@ualg.pt (A.R.); 3 REQUIMTE/LAQV: Laboratório de Farmacognosia, Departamento de Química, Faculdade de Farmácia, Universidade do Porto, Rua de Jorge Viterbo Ferreira n. 228, 4050-313 Porto, Portugal; pandrade@ff.up.pt (P.B.A.); mfgfernandes@gmail.com (F.F.)

**Keywords:** marine invertebrates, marine biotechnology, natural antioxidants, nutritional profile, carotenoids, fatty acids

## Abstract

**Simple Summary:**

Marine invertebrates are a rich source of natural products with multiple commercial applications. However, little is known about the biotechnological potential of *Sabella spallanzanii* and *Microcosmus squamiger.* Aimed at filling this gap, this work evaluated such species for biochemical, antioxidant and metal chelating properties. Both species had interesting nutritional profiles in terms of crude protein and fat, among others. Regarding amino acids, *S. spallanzanii* and *M. squamiger* were found to be rich in arginine and taurine, respectively, while the most abundant minerals were sodium, calcium, and potassium, in both species. Methanol extracts of both species had antioxidant and metal chelating capacity. Fucoxanthinol and fucoxanthin were the major carotenoids in the *M. squamiger* dichloromethane extract. The fatty acid (FA) profile was strongly influenced by the type of extract. Saturated FA were higher than unsaturated ones in methanol extracts, while unsaturated FA prevailed in the dichloromethane extracts. Low *n*-6/*n*-3 ratios were obtained. Our results suggest both species as sustainable sources of bioactive products with several applications, for example, as food ingredients, as fish feed, and as ingredients for veterinary products.

**Abstract:**

*Sabella spallanzanii* and *Microcosmus squamiger* were profiled for proximate composition, minerals, amino acids, fatty acids (FA), carotenoids, radical scavenging activity on the 2,2-diphenyl-1- picrylhydrazyl (DPPH) radical, oxygen radical absorbance capacity (ORAC) and iron and copper chelating properties. *Microcosmus squamiger* had the highest level of moisture and crude protein, *S. spallanzanii* was enriched in crude fat and ash. Both species had similar levels of carbohydrates and energy. There was a prevalence of arginine and glycine in *S. spallanzanii*, and of taurine in *M. squamiger*. The most abundant minerals in both species were Na, Ca, and K. The methanol extract of *S. spallanzanii* had metal chelating properties towards copper and iron, while the methanol extract of *M. squamiger* was able to chelate copper. *M. squamiger* extracts had similar ORAC values. Fucoxanthinol and fucoxanthin were the major carotenoids in the *M. squamiger* dichloromethane extract. Saturated FA were more abundant than unsaturated ones in methanol extracts, and unsaturated FA prevailed in the dichloromethane extracts. Palmitic acid was the predominant FA in methanol extracts, whereas eicosapentaenoic (EPA) and dihomo-γ-linolenic acids were the major compounds in dichloromethane extracts. Low *n*-6/*n*-3 ratios were obtained. Our results suggests that both species could be explored as sources of bioactive ingredients with multiple applications.

## 1. Introduction

More than 40,000 marine natural products (MNPs) have been identified, from several organisms, including macro- and micro-algae and different taxa of invertebrates, such as sponges, cnidarians, tunicates, mollusks, echinoderms, and bryozoans [1]. Research on MNP has focused on marine invertebrates, which produce a plethora of primary and secondary metabolites vital to complex adaptation mechanisms to the marine life conditions, including defense from predators, competitors, and infective agents, coping with salinity and UV radiation, and inter-individual signalization [2]. Such metabolites belong to different classes (e.g., polyketides, terpenoids, alkaloids, lipids), exhibit highly relevant pharmacological properties, (e.g., antioxidant, anti-inflammatory, anti-tumoral), and have multiple industrial applications, for example, in the food, cosmetic and aquaculture sectors.

**Figure 1:**
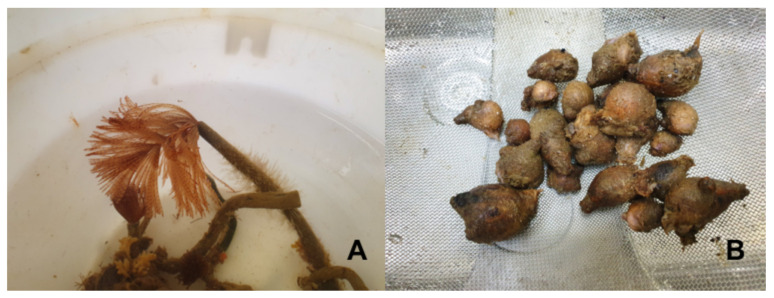
Collected specimens of *Sabella spallanzanii* (**A**) and *Microcosmus squamiger* (**B**). (Photo by Yu Lun-Pan).

The Mediterranean fan worm *Sabella spallanzanii* (Gmelin, 1791) (Phylum: Annelida, Class: Polychaeta) (Figure 1A) is a native species from the temperate zone of the Mediterranean Sea and Southern European Atlantic coast and is an invasive species in Australia and New Zealand [3,4,5]. It is commonly found on the sediment substrate or artificial hard surfaces to a depth of 30 m and is part of the diet of several fish species and invertebrates, including cephalopods [6]. Moreover, *S. spallanzanii* is a suspension feeder and can filter high quantities of water and accumulate metals in its tissues and is therefore considered a suitable biomonitor for metal pollution in marine areas [4,7,8,9]. Marine invertebrates, including Polychaeta, are widely used as feed ingredients, especially in diets for shrimp [6]. *Sabella spallanzanii* is a potential candidate for bioremediation of aquaculture waste [6,7], and a potential resource for the fish feed industry due its biochemical profile, especially its high protein and gross energy levels, mineral and fatty acids profile, high concentration of glycosaminoglycan (GAGs), and high levels of amino acids (e.g., glutamic acid, arginine, glycine) that are considered as attractants for different commercial fish species [6]. *Microcosmus squamiger* (Michaelsen, 1927) (Phylum: Chordata, Class: Ascidiaceae) (Figure 1B) is a cosmopolitan sessile species native to Australia, and is biologically invasive in several areas, including the Iberian Peninsula Atlantic, Mediterranean, North Africa Atlantic, Red Sea, East Pacific, and Indian ocean [10,11,12,13,14,15,16,17]. Moreover, recent findings show that *M. squamiger* has spread from the western Mediterranean to the eastern Mediterranean [18], and to the west coast of North America [19]. In its natural condition, *M. squamiger* inhabits hard littoral substrates in a depth up to 10 m [10], while in the introduced range, it can be found not only in high densities in open coastal habitats where it affects native biota, but also in artificial structures, such as shipping harbors, where it forms dense clumps covering the available substratum [20,21], and also competing for space in cultured bivalves [17]. Information regarding the biochemical properties of *M. squamiger* is limited to the composition of the tunic of this species [22], and to the levels of moisture, lipids, protein, and ash [23] and metals [24] of individuals collected in India. A closely related species, *M. exasperates*, has a balanced nutritional profile, and contains several bioactive molecules, including vitamins (e.g., D3, K, riboflavin), phenolic compounds (e.g., gallic acid, ferulic acid) and flavonoids (e.g., rutin, quercetin) [25].

Invasive species have severe economic and ecological impacts [26,27,28,29]. There are several strategies to control invasive species, and one of the most effective is their use as sources of products with commercial applications [27], which is being exploited for the control of different invasive macroalgae species [27,28,29]. In this context and having in mind existing information regarding the biochemical properties of *S. spallanzanii* and *M. squamiger*, and the fact that both species can co-exist in the same location, this work aimed to determine if both species could be exploited in the context of blue biotechnology as sources of nutritional and bioactive compounds. For this purpose, we have focused on the nutritional profile of the animal’s biomass, including proximate composition, minerals, and amino acids levels, and on the fatty acids (FA), carotenoid contents, and on the antioxidant properties of methanol and dichloromethane extracts obtained from said biomass.

## 2. Materials and Methods

### 2.1. Chemicals

All reagents and solvents were of analytical grade. Sodium acetate, 2,2-diphenyl-1-picrylhydrazyl (DPPH), potassium dihydrogen phosphate and pure standards of amino acids were purchased from Sigma-Aldrich (Lisbon, Portugal). 2,20-azobis (2-amidinopropane) dihydrochloride (AAPH) and 6-hydroxy-2,5,7,8-teramethylchroman-2-carboxylic acid (Trolox) were acquired from Acros Organics (Geel, Belgium). Panreac Química (Castellar Del Valles, Spain) provided fluorescein and nitric acid. Dimethyl sulfoxide (DMSO), ethylenediamine tetraacetic acid (EDTA), copper (II) sulfate, and additional chemicals were obtained from VWR International (Leuven, Belgium). A mixed standard solution of fatty acid methyl esters (FAME) (CRM47885) was obtained from Supelco (Bellefonte, PA, USA). Chloroform, iso-octane, boron trifluoride (BF3)-methanol solution, potassium hydroxide (KOH) and anhydrous sodium sulphate were purchased from Sigma-Aldrich (St. Louis, MO, USA). Methanol was acquired from VWR (Fontenay-sous-Bois, France). Standards of fucoxanthin (≥95.0%) and β-carotene (≥95.0%), and methyl tert-butyl ether (MTBE) were purchased from Sigma-Aldrich (St. Louis, MO, USA). Standards of astaxanthin (95.0%), antheraxanthin (≥95.0%), zeaxanthin (97.0%), ε, ε-carotene, α-carotene and γ-carotene were obtained from CaroteNature (Lupsinggen, Switzerland). Fucoxanthinol (≥95.0%) was from FLuka (Buchs, Switzerland). Methanol (MeOH) Lichrosolv was acquired from Merck (Darmstadt, Germany).

### 2.2. Sampling

Adult specimens of *S. spallanzanii* and *M. squamiger* (Figure 1) were hand-collected as a bulk from the floating dock in Faro, Portugal (37°00′15.4″ N 7°59′16.8″ W) in January 2020 and transported to the Centre of Marine Sciences (CCMAR, University of Algarve, Gambelas Campus). Species identification was conducted by traditional taxonomic characters by Dr. Carlos Afonso from the Fisheries, Biodiversity and Conservation research group from CCMAR. The polychaetes and ascidians were separated, and samples were rinsed with tap water, followed by distilled water, to remove contaminants. Samples were freeze-dried, milled, and stored at −20 °C.

### 2.3. Proximate Composition

Moisture was measured by drying the fresh biomass at 60 °C until constant weight, while ash was determined by incineration in a muffle furnace at 525 °C for 5 h. The crude protein content was determined by the elemental analysis of nitrogen (N), in a combustion analyzer. The N value was then multiplied by the conversion factor (6.25) to determine the crude protein [30]. Total lipids were determined by the modified method from Bligh and Dyer, as described previously [31]. Carbohydrates were calculated by difference. The gross energy (GE) was calculated using the Atwater general factor system: GE (MJ) = 0.017878 × (g protein) + 0.037765 × (g lipid). Results were presented as percentages for moisture, ash, protein, and fat. GE was represented as MJ/kg of dry weight biomass (dw).

### 2.4. Amino Acids

The tissues levels in free amino acids were determined by ultra-high performance liquid chromatography (UPLC). In brief, freeze dried samples were homogeneized in hydrogen chloride (HCl, 0.1 M) on ice, and centrifuged (1500× *g*, 4 °C, 15 min). The supernatant was collected and deproteinized by centrifugal ultrafiltration (10 kDa cut-off, 2500× *g* at 4 °C for 20 min). Samples were pre-column derivatized with the Waters AccQ fluor reagent (6-aminoquinolyl-N-hydroxysuccinimidyl carbamate) using the AccQ Tag method (Waters, USA). Analyses were performed by UPLC on a Waters reversed-phase amino acid analysis system, using norvaline as an internal standard. Amino acids were identified by comparing their retention times with those of standard mixtures and pure standards. Instrument control, data acquisition and processing were achieved using Waters Empower software (Milford, MA, USA).

### 2.5. Minerals

Dried biomass (25 mg) was digested with nitric acid (HNO_3_, 6 mL) and hydrogen peroxide (H_2_O_2_, 2 mL) using a CEM Discover microwave synthesizer (Matthews, NC, USA). The resulting solutions were diluted in ultrapure water to give sample solutions in serial concentrations. The concentrations of minerals in the sample solutions were analyzed using Agilent 4200 microwave plasma-atomic emission spectroscopy (MP-AES) (Santa Clara, CA, USA). The calibration curve was obtained by referring to the multielement standard solution. Agilent MP Expert software (Santa Clara, CA, USA) was used to subtract the background signal from the analytical signal and data acquisition.

### 2.6. Preparation of the Extracts

Dried biomass (1 g) was separately extracted with dichloromethane or methanol (40 mL) during 30 min in an ultrasound bath (USC–TH, VWR, Portugal), with a capacity for 5.4 L, frequency of 45 kHz, a supply of 230 V and a tub heater of 400 W, at room temperature (RT). The extracts were then filtered (Whatman n°4) and evaporated under controlled temperature (≤45 °C) and pressure in a rotary evaporator (BUCHI R -210, Flawil, Switzerland). The obtained dried extracts were resuspended in DMSO for the carotenoid’s determination, and for antioxidant activity and metal chelating assays, and in iso-octane for the fatty acids analysis.

### 2.7. Chemical Characterization of the Extracts

#### 2.7.1. FA Determination

For FA characterization, the extracts were subjected to an alkaline hydrolysis to obtain free fatty acids and derivatized to obtain the corresponding methyl esters (FAME), being then analysed by gas chromatography (GC-FID), as described previously [32]. Samples (1 μL) were injected in triplicate, and the levels of FAME present were obtained from calibration curves of the respective standards using at least six concentrations of the analytes, according to the range of concentrations found in the samples. The limit of detection (LOD) and limit of quantification (LOQ) (Table S1, Supplementary Materials) were determined from the calibration curves, according to the following formula:

LOD = 3 × SD(1)

LOQ = 10 × SD(2)

where SD is the residual standard deviation of the linear regression (Table S1, Supplementary Materials).

#### 2.7.2. HPLC-DAD Analysis for Carotenoid Determination

The carotenoid contents of the extracts were determined by liquid chromatography (HPLC-DAD), as described elsewhere [32,33]. Identification of the compounds was achieved by comparing their retention times and UV-Vis spectra in the range of 200–700 nm with those of pure standards. Peak purity was assured by the software contrast facilities and pigment quantification was accomplished by the absorbance verified in the chromatograms (at 450 nm) in relation to external calibration curves, which were obtained by using, at least, six different concentration levels of the standards, according to the array of concentrations detected in the samples. LOD and LOQ were determined from the calibration curves (Table S2, Supplementary Materials), according to Equations (1) and (2).

### 2.8. In Vitro Radical Scavenging Activity (RSA) of the Extracts

#### 2.8.1. RSA on DPPH

The RSA towards DPPH was determined according to Rodrigues et al. [34] on samples ranging from 0 to 10 mg/mL. Absorbances were read at 492 nm in a microplate reader (Biochrome EZ400), and RSA was calculated in relation to the blank containing DMSO. Results were expressed as half maximal inhibitory concentration (IC_50_ values), when possible. Butylated hydroxytoluene (BHT) was used as the positive control at 1 mg/mL.

#### 2.8.2. Oxygen Radical Absorbance Capacity (ORAC) Assay

ORAC was measured following the method described by Gillespie et al. [35] on extracts diluted to adequate concentrations in relation to Trolox. The decrease in fluorescence was established by reading fluorescein excitation at 485 nm and emission at 530 nm, for a period of 90 min, each minute, in a microplate reader (Tecan Infinite M200 multi detection plate reader). ORAC results were calculated using the area under the curve (AUC) and results were expressed as mmol of Trolox equivalents per 100 g of dry weight (mmol TE/100 g).

### 2.9. In Vitro Metal Chelating Properties of the Extracts

#### Copper (CCA) and Iron (ICA) Chelating Activity

CCA and ICA were determined by the methods described by Rodrigues et al. [34], on the extracts at concentrations up to 10 mg/mL. EDTA (1 mg/mL) and DMSO were used as positive and negative controls, respectively. Absorbance was measured at 620 nm for copper and 562 nm for iron, in a microplate reader (Biochrome EZ400). Results were calculated in relation to the negative control and expressed as IC_50_ values whenever possible.

### 2.10. Statistical Analyses

Statistical analysis was performed using SPSS Statistics software v. 22 (IBM SPSS Statistics for Windows, IBM Corp., Armonk, NY, USA). Analyses were conducted at least in triplicate and expressed as mean ± standard error of the mean (SEM) or as mean ± standard deviation. A one-way analysis of variance (ANOVA) with Tukey’s HSD post hoc test (*p* < 0.05) was used to look for statistically significant differences among results. Differences amongst samples were considered significant if *p* values were equal or inferior to 0.05. The IC_50_ values were determined through data sigmoidal fitting in the GraphPad Prism v. 5.0 software (San Diego, CA, USA).

## 3. Results

### 3.1. Proximate Composition

The proximate composition and energetic value of *S. spallanzanii* and *M. squamiger* can be found in Table 1. The moisture, ash, and total levels of protein and lipid of *S. spallanzanii* were 70.7%, 53.1%, 47.2% and 11.7%, while those of *M. squamiger* were 88.9%, 36.2%, 53.9% and 5.3%. The calculated gross energy was 12.8 MJ/kg for *S. spallanzanii*, and 11.6 MJ/kg for *M. squamiger*.

### 3.2. Amino Acids

A total of 26 free amino acids (FAA) were identified, representing 131.76 mg/g (dw) in *S. spallanzanii* and 52.42 mg/g (dw) in *M. squamiger* (Table 2). The predominant amino acid in *S. spallanzanii* was arginine (68.5 mg/g, dw), corresponding to 2008 mg/100 g (wet weight, ww), followed by glycine (49.3%), while the content of the remaining FAA varied from 0.03 mg/g (tryptophan) to 2.30 mg/g (taurine). The most abundant FAA in *M. squamiger* was taurine (26.64 mg/g, dw, corresponding to 296 mg/100 g, ww), while the levels of the other amino acids ranged from 0.15 mg/g (cysteine) to 5.15 mg/g (proline).

### 3.3. Minerals

The mineral content of *S. spallanzanii* and *M. squamiger* is summarized in Table 3. The most abundant macroelements in *S. spallanzanii* were sodium (Na, 18.65 mg/g, dw), calcium (Ca, 17.09 mg/g, dw), and potassium (K, 15.14 mg/g, dw), while the most abundant microelements in this species were aluminium (Al, 13.10 mg/g, dw), iron (Fe, 12.12 mg/kg, dw) and vanadium (V, 11.6 mg/kg, dw). In *M. squamiger*, the dominant macroelements were Na (36.24 mg/g, dw), Ca (14.29 mg/g, dw) and K (11.88 mg/g, dw), and the most abundant microelements were V (4.63 mg/g, dw), Al (4.60 mg/g, dw) and iron (Fe, 4.42 mg/g, dw). Thorium (Th) was detected in trace amounts (*S. spallanzanii*, 0.28 mg/g, dw; *M. squamiger*, 0.48 mg/g, dw).

### 3.4. Chemical Profile of S. spallanzanii and M. squamiger Extracts

#### 3.4.1. FA

**Figure 2:**
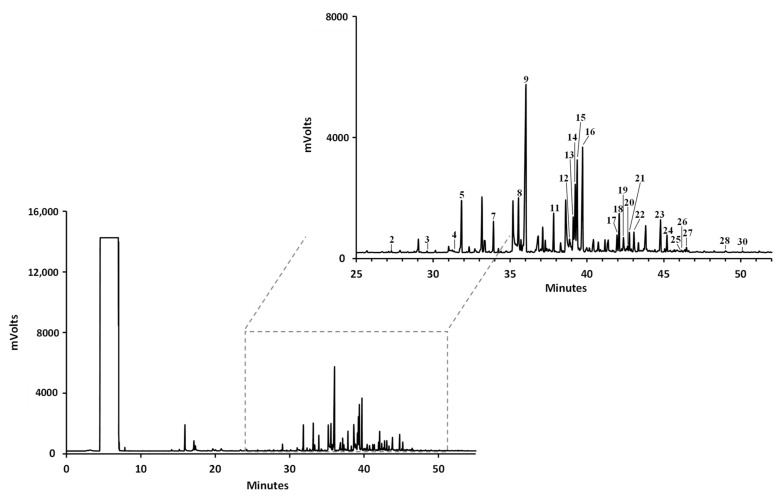
Representative GC-FID chromatogram of the fatty acid profile of *Microcosmus squamiger* methanol extract. (1) Undecylic acid (C_11:0_); (2) lauric acid (C_12:0_); (3) tridecyclic acid (C_13:0_); (4) myristoleic acid (C_14:1*n*-5c_); (5) myristic acid (C_14:0_); (6) cis-10-pentadecenoic acid (C_15:1*n*-5c_); (7) pentadecyclic acid (C_15:0_); (8) palmitoleic acid (C_16:1*n*-7c_); (9) palmitic acid (C_16:0_); (10) cis-10-heptadecenoic acid (C_17:1*n*-7c_); (11) margaric acid (C_17:0_); (12) γ-linolenic acid (C_18:3*n*-6c_); (13) linoleic acid (C_18:2*n*-6c_); (14) oleic acid (C_18:1*n*-9c_); (15) elaidic acid (C_18:1*n*-9t_); (16) stearic acid (C_18:0_); (17) arachidonic acid (C_20:4*n*-6c_); (18) eicosapentaenoic acid (C_20:5*n*-3c_); (19) homo-γ-linolenic acid (C_20:3*n*-6c_); (20) eicosadienoic acid (C_20:2*n*-6c_); (21) α-linolenic acid (C_18:3*n*-3c_); (22) arachidic acid (C_20:0_); (23) heneicosylic acid (C_21:0_); (24) docosahexaenoic acid (C_22:6*n*-3c_); (25) linoelaidic acid (C_18:2*n*-6t_); (26) erucic acid (C_22:1*n*-9c_); (27) behenic acid (C_22:0_); (28) tricosylic acid (C_23:0_); (29) nervonic acid (C_24:1*n*-9c_); (30) lignoceric acid (C_24:0_).

Free FA qualitative and quantitative profiles of methanol and dichloromethane extracts obtained from *M. squamiger* and *S. spallanzanii* were achieved by GC-FID, after saponification and derivatization to their respective methyl esters; results are summarized in Table 4 and Figure 2. Thirty free fatty acids, containing between 11 and 24 carbon atoms, with different degrees of unsaturation, were identified in both species (Table 4). The quantification revealed a total FA concentration ranging from 19.09 µg/mg to 459.25 µg/mg (dw). The highest FA amounts were found in *S. spallanzanii* dichloromethane extract. The FA profiles were strongly influenced by the type of extract. Total FA content was considerably higher in *S. spallanzanii* and *M. squamiger* dichloromethane extracts than those of methanolic extracts (459.25 and 251.34 µg/mg against 21.56 and 20.09 µg/mg (dw), respectively). Although their absolute FA content was much lower than that found in dichloromethane extracts, methanol extracts of both species showed a higher content of saturated FA than unsaturated ones. Palmitic acid (C_16:0_) (9) was the major fatty acid in *S. spallanzanii* and *M. squamiger* methanol extracts (accounting for 24.2 and 33.1% of total fatty acids) and the most representative saturated FA in *S. spallanzanii* and *M. squamiger* dichloromethane extracts representing ca. 19.5 and 33.1% of total FA. On the contrary, almost 70% of the total content of FA found in dichloromethane extracts are unsaturated, distributed by more than 20% monosaturated FA (MUFA) and almost 50% polyunsaturated FA (PUFA). cis-9-hexadecenoic acid (C_16:1*n*-7c_) (8) and trans-9-octadecenoic acid (C_18:1*n*-9t_) (15) were the more representative MUFA in both extracts. The main PUFA detected were C_18_ and C_20_ FA and the concentrations of C_20_ PUFA were higher than those of C_18_ PUFA. cis-5,8,11,14,17-Eicosapentaenoic (C_20:5*n*-3c_) (EPA) (18) and cis-8,11,14-eicosatrienoic (C_20:3*n*-6c_) (19) acids, *n*-3 and *n*-6 series, respectively, were the major compounds found in *M. squamiger* and *S. spallanzanii* dichloromethane extracts, accounting for 22.4 and 27.2% of total FA, respectively. In addition of EPA (18), considerable amounts of cis-4,7,10,13,16,19-docosahexaenoic acid (C_22:6*n*-3c_) (DHA) was observed in dichloromethane extracts, representing ca. 15 and 5% of total FA in *M. squamiger* and *S. spallanzanii*, respectively.

#### 3.4.2. Carotenoids

**Figure 3:**
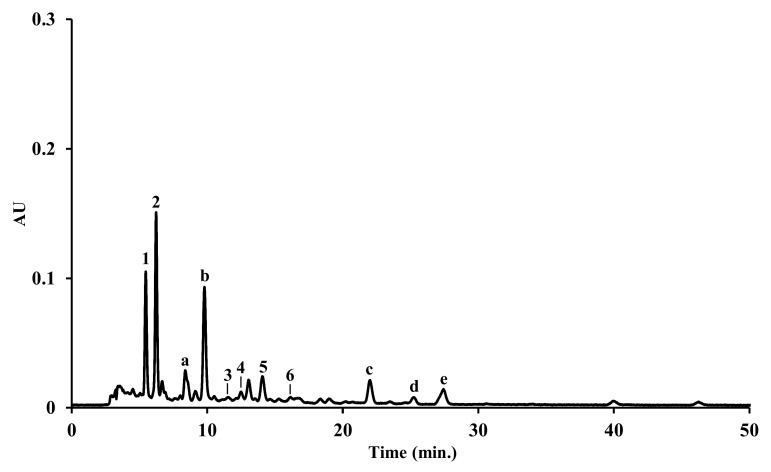
HPLC-DAD carotenoid profile of the dichloromethane extract from *Microcosmus squamiger*. Identity of compounds as in Table 5.

The carotenoid composition of the extracts is depicted in Table 5 and Figure 3. Six xanthophylls were identified in *M. squamiger* dichloromethane extract; however, no carotenoids were detected in the *M. squamiger* methanolic extract, nor in the *S. spallanzanii* extracts (dichloromethane and methanolic). In addition to fucoxanthinol (1), two other fucoxanthin-related compounds (a and b) were found in *M. squamiger* dichloromethane extract; however, their identification was not achieved. Additionally, the presence of three carotenes whose identity could not be defined was observed. The injection of α-carotene, β-carotene, ε-carotene, and γ-carotene, at same chromatographic conditions, allows us to ensure that it is none of these compounds. Fucoxanthinol (1) and fucoxanthin (2) were the major compounds.

### 3.5. Antioxidant Properties of the Extracts

In this work, dichloromethane and methanol extracts from *S. spallanzanii* and *M. squamiger* were evaluated for the first time for antioxidant properties, by four complementary *in vitro* methods, targeting free radicals and redox metals, namely copper (Cu) and iron (Fe), and results are summarized in Table 6. In *S. spallanzanii*, the extraction yields for dichloromethane and methanol extracts were 5.06 and 6.32%, respectively, and the IC_50_ values were not reached at the maximum concentration tested (10 mg/mL) for the dichloromethane extract. However, the methanol extract of *S. spallanzanii* exhibited metal chelating properties towards both metals with IC_50_ values of 1.7 mg/mL and 4.1 mg/mL for Cu and Fe, respectively. In *M. squamiger*, the extraction yields for dichloromethane and methanol extracts were 2.42 and 15.6%, respectively, and, similar to observations in *S. spallanzanii*, the application of the dichloromethane extract did not allow us to reach the IC_50_ values, up to 10 mg/mL, in all the assays. However, the methanol extract was able to chelate Cu ions, with an IC_50_ value of 7.6 mg/mL.

**Figure 4:**
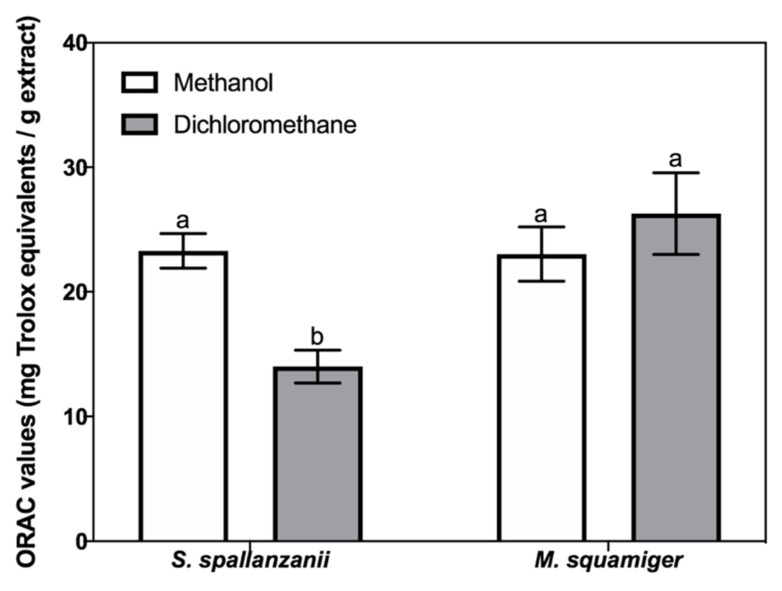
Antioxidant activity (mg trolox equivalents TE/g) determined by the oxygen radical absorbance capacity (ORAC) assays in dichloromethane and methanol extracts of *Sabella spallanzanii* and *Microcosmus squamiger.* For the same species, collumns labelled with different letters re significantly different (*p* < 0.05).

All samples showed antioxidant activity in the ORAC assay (Figure 4). Regarding *S. spallanzanii*, the methanol extract had a higher activity (23.3 ± 3.1 mg TE/g extract) than the dichloromethane extract (14.0 ± 3.0 mg TE/g extract). In *M. squamiger*, similar ORAC values were obtained for the methanol (23.0 ± 4.9 mg TE/g) and dichloromethane extract (26.3 ± 7.3 mg TE/g).

## 4. Discussion

Blue Biotechnology is considered by the European Union (EU) as one of the bases for the blue growth agenda, which aims to guarantee the sustainable use of marine resources [36]; efforts are being applied to identify new marine species as sources of products with commercial applications, such as medical drugs, foodstuffs, feed, and cosmetics [37,38,39,40,41]. The identification of such uses for invasive species could contribute for their sustainable use and control [27,28,29,42]. This work focused on the biochemical properties of *S. spallanzanii* and *M. squamiger*, aiming for their biotechnological valorisation. Both species are invasive and can co-exist in their habitat. *Sabella spallanzanii* belongs to the Polychaeta group, the major among Annelida, occupying almost every area of the marine environment [39]. Despite its abundance and ecological relevance, research on the biotechnological uses of marine Polychaeta is more recent than those focusing on terrestrial or continental water annelids [39]. *Microcosmus squamiger* is an Ascidian, a group of marine invertebrates considered an important source of secondary metabolites with important biological properties, such as anti-fouling and anti-tumoral [43].

Aiming to gain an insight into the nutritional properties of both species, samples were profiled for proximate composition, specifically moisture, ash, protein, lipids, and energetic value. The moisture level of *S. spallanzanii* was lower than the value reported for the same species collected in the Southern Adriatic Sea, Italy (79.3%) [6], which may be related with the fact that, in the latter study, the leathery tube was removed before analysis. The moisture content of *M. squamiger* was similar to the value reported for the same species collected in the Palk Bay region, Southeast coast of India [23]. Recent studies have shown the potential of marine invertebrates, including annelid worm and tunicates, as potential sources of alternative fish meal in view of the current trend in aquaculture expansion and the consequent growing need to identify new sources for fish feed ingredients [6,20,44]. Therefore, one of the possible uses of biomass from *S. spallanzanii* and *M. squamiger* could be as ingredients in fish meal. The moisture content is important when considering ingredient storage and in the feed manufacturing process, such as extrusion; however, it does not necessarily negatively impact the final moisture content of the feed [45]. The ash levels of *S. spallanzanii* and *M. squamiger* were higher than those reported for the same species [6,24], which may be related to the fact that, in the present work, the whole organisms were analysed, while previous studies evaluated only muscle samples. High ash levels have implications in fish feed: a fish meal with a high ash content usually results in a faster transit rate, which leads to increased growth feed intake and growth, but also to poor feed efficiency [46].

When considering the nutritional value of a species for use in animal nutrition, macronutrients such as proteins and lipids are the most important ingredients [47], since they are essential for maintaining growth, reproduction, and physiological functions of the target animals [48]. A high-quality fish meal should contain up to 72% of protein by weight [49]. The high protein level of *S. spallanzanii* determined in this work is lower than the value reported for the same species (54.8%, dw) [6], while the *M. squamiger* protein content was higher than that reported for the same species [24]. Determined levels are lower than the recommended protein contents of fish meals, but higher than those reported for some edible marine invertebrates, such as mussel (*Mytilus* sp., 37.9%) and Japanese sea cucumber (*Stichopus japonicus* Selenka, 1867, (4.74%) [50,51], thus suggesting its potential as a protein source for human consumption. Moreover, protein from marine invertebrates exhibit an array of highly relevant biological properties, including anticancer, antioxidant and tissue regeneration assets, and, therefore, are highly valued by the nutraceutical and biomedical industries [40]. Both species had low crude fat levels, which is in accordance with previous results in *S. spallanzanii* (8% with seasonal variation) [6] and *M. squamiger* [24]. Low crude fat is common in different marine invertebrates, as, for example, the polychaete *Nereis diversicolor* (O.F. Müller, 1776) [25], and several ascidian species collected from the southeast coast of India [23]. Such fat levels correspond to fatty and semi fat fish and are lower to the total lipids commonly present in commercial fish feed (10–25%) [48]. The calculated gross energy for *S. spallanzanii* was lower than the results reported for the same species (4.8 kcal/g, corresponding to 20.4 MJ/kg [6].

Amino acids (AAs) are the basic building blocks of proteins and play an essential role in basic physiological performance by intervening in the bioavailability of nutrients and gene expression. The level of total free amino acids was higher in *S. spallanzanii*, and, in this species, the most abundant amino acid was arginine, which is consistent with the findings of Stabili et al. [6], in the same species, and similar to values reported for several foods, including beef [52]. Arginine is an essential AA to humans and can be provided by lean meats, nuts, seeds, and leguminous. It plays a major role in protein synthesis, improves endothelial and immune function, and reduces oxidative stress and vasodilation by increasing the production of nitric oxide (NO) [53,54]. Arginine has an important role in vascular function and skeletal muscle maintenance and is a popular nutritional supplement since it has beneficial effects on athletic performance and health in general [55]. The intake of glycine is also linked with a reduction of total cholesterol levels in serum [56]. The predominance of arginine is especially relevant when considering the use of *S. spallanzanii* as a fish meal ingredient, because arginine is an indispensable amino acid required by all fish species [57]. For example, a deficiency of arginine in diet resulted in reduced growth and low feed efficiency in Japanese flounder (*Paralichthys olivaceus* Temminck & Schlegel, 1846), milkfish (*Chanos chanos* Forsskål, 1775) and Atlantic salmon (*Salmo salar* Linnaeus, 1758) [58,59,60]. In channel catfish (*Ictalurus punctatus* Rafinesque, 1818), high mortality and fin erosion were reported because of arginine deficiency [61]. Besides, glycine is the major constituent of collagen and elastin [62]. Collagen is responsible for the strength, rigidity, and flexibility of connective tissues and is essential for growth, development, and overall animal health [63]. Elastin is also structurally important in connective tissue, thus giving the elastic properties to vertebrate organs and tissues [64].

The second most abundant amino acid in *S. spallanzanii* was glycine, which can be synthesized by the animals through interconvertion from serine in the liver and kidneys by tetrahydrofolate-dependent hydroxymethyltransferase [65]. However, the energy cost may result in suboptimal growth [39]. Xie et al. [66] showed that dietary supplementation with 0.3% of glycine enhanced growth rate in juvenile whiteleg shrimp (*Litopenaeus vannamei* Boone, 1931). Furthermore, a supplementation with 0.5% of glycine resulted in the improvement of weight gain, antioxidative capacity, and immunity in Nile tilapia (*Oreochromis niloticus* Linnaeus, 1758) [67]. Besides, the sweet flavour of glycine stimulated the food intake of giant tiger prawn (*Penaeus monodon* Fabricius, 1798) [68]. Therefore, our results suggest that *S. spallanzanii* could have potential use as a natural attractant, with the potential to replace synthetic compounds, in line with the requirements of the modern trend for organic fish production [6]. Attractants are compounds or ingredients that, when added to the feed, boosts its taste and acceptableness by fish, and are widely used in several sectors of animal production, including aquaculture [6,69]. The price of attractants is higher than that of fish meal, and are already used in fish feeds, especially when fish meal is replaced by diets rich in plant-based protein [6].

Taurine was identified as the dominant FAA in *M. squamiger* in higher levels than those reported for important sources of taurine, including cod (*Gadus* spp.) fillet (120 mg/100 g, ww), saithe (*Pollachius virens*), and peeled shrimps (220 mg/100 g, ww) [70]. Taurine (a sulfur-containing β-amino acid) is indicated as a functional feed additive due to its importance for the improvement of fish performance, immunity, and intestinal health [71,72,73]. The benefits of taurine supplementation are species-specific in relation to the levels in the feed and feeding duration [73]. Gaylord et al. [74] showed that supplementation of taurine improved the growth, feed conversion ratios, protein retention efficiencies and energy retention efficiencies in rainbow trout (*O. mykiss*). Moreover, the weight gains, feed conversion ratios and protein efficiency ratios of giant tiger prawn (*P. monodon*) were higher when taurine was included in the fish diet [75]. Taurine deficiency resulted in green liver syndrome and poor growth performance in Japanese amberjack (*Seriola quinqueradiata* Temminck & Schlegel, 1845) and red seabream (*Pagrus major* Temminck & Schlegel, 1843) [76,77]. The ability to synthetise taurine was detected in some species, such as Japanese flounder (*P. olivaceus*) and rainbow trout (*O. mykiss*); however, it was not observed in others, as, for example, Japanese amberjack (*S. quinqueradiata*), Atlantic bluefin tuna (*Thunnus thymus* Lineu, 1758) and skipjack tuna (*Katsuwonus pelamis* Linnaeus 1758) [78]. When the endogenous fish production is absent or insufficient to meet the physiological needs, taurine supplementation is required to avoid deficiency [79]. It should be mentioned that alternative protein sources for fishmeal, such as soybean meal, contain very low taurine levels [65]. Supplementation of synthetic taurine in fish diets is allowed in Europe [80]. However, in the USA, taurine supplementation is only allowed if it is added indirectly to the fishmeal through a feed ingredient naturally rich in that element [79].

Moreover, taurine is a physiologically essential nutrient for humans, especially infants and children, and plays major roles in human physiology and nutrition, including the facilitation of intestinal absorption of dietary lipids and cholesterol elimination, stabilization of cell membranes, besides exhibiting antioxidant and anti-inflammatory properties, among others [81,82]. Humans have a very low ability to synthesize taurine, when compared to other animals (e.g., livestock and poultry), and it is abundant in animal-source foods (such as beef) but almost absent in plants [83,84]. Therefore, vegetarians and vegans are at increased risk for taurine deficiency since the precursors for taurine synthesis (methionine and cysteine) are present at low levels in most proteins of plant origin [82]. Taurine has been used for 45 years as a dietary supplement to improve health in humans and animal models of metabolic syndrome [82,85]. The use of taurine supplementation is especially important in type-2 diabetic patients, since they exhibit a 25% lower concentration of taurine in plasma than normal subjects [86]. Moreover, recent research has shown that it has a protective effect against various xenobiotics, mainly due to its anti-oxidative, anti-inflammatory, and membrane-stabilizing properties, and is regarded as a promising dietary supplement for the treatment of liver diseases [87]. Besides its importance to humans, taurine supplementation is also highly relevant in veterinary practice. For example, taurine is not an essential amino acid in dogs, since taurine precursors (cysteine and methionine) are used by the liver and the central nervous system to synthesize taurine. However, taurine supplementation can revert dilated cardiomyopathy, a common dog disease, since it is linked to reduced plasma taurine levels [88,89,90,91]. Overall, our results suggest *M. squamiger* as a source of taurine for human and veterinary uses, and of fishmeal taurine-rich ingredients.

Minerals have key metabolic roles in all organisms and are therefore important ingredients in formulations used in different commercial areas, for example, as food and feed ingredients. The most abundant minerals in *S. spallanzanii* and *M. squamiger* were Na, Ca, and K. The Na and Ca levels of *S. spallanzanii* were lower than those detected in the same species, by other authors [6]. Besides being a structural constituent of bones, exoskeleton, teeth and scales, Ca is a cofactor for enzymatic processes and is crucial in the conservation of cell membrane integrity, blood clotting, muscle contraction, bone mineralization, adequate nerve impulse transmission and osmoregulation. Seawater contains a significant amount of dissolved Ca, and fish can absorb it from the surrounding water to fulfil their metabolic requirements [92]. The level of K in *S. spallanzanii* was higher than the one detected in the same species, collected elsewhere [6]. This element is an essential component of the fish diets since it is crucial for skeletal development and intermediary metabolism [93]; however, since P supplementation in fish feed is an important source of eutrophication, its supplementation must be a balance between the P requirements of fish and the possible environmental impacts. If considering *M. squamiger* biomass as a potential fishmeal ingredient, its Ca levels could be relevant, since several studies have shown that adequate dietary Ca supplementation has beneficial effects on the performance of several fish species, for example, redlip mullet (*Liza haemmatocheila* Temminck & Schlegel, 1845) [94].

The most abundant micro elements in *S. spallanzanni* were aluminium Al, Fe, and V, while in *M. squamiger* they were V, Al, and Fe. The Fe level in *S. spallanzanii* was higher than the level detected in the same species [6]. Iron is an important micronutrient for teleost fish, is an integral component of proteins, participates in cellular respiration and oxygen transfer, and is routinely added to fish feeds in aquaculture [95]. Vanadium was also detected, although in lower amounts (0.56 ppm) in *M. squamiger* collected in the southeast coast of India [24]. Ascidians accumulate V in their blood cells, vanadocytes [96], and the toxicity and essentiality of this element are still inconclusive [97]. No toxic elements were detected in both species, except for Th, which was detected in trace amounts. Thorium is a radioactive metal with natural occurrence in the Earth’s crust, in minerals such as thorite and monazite [98]. Radioactive elements emitting alpha particles are of high concern since they are highly radiotoxic to the body. Thorium has three alpha-emitting radioisotopes: ^228Th^ (T_1/2_  =  1.91 y), ^230Th^ (T_1/2_  =  7.54 × 10^4^ y), ^232^Th (T_1/2_  =  1.4 × 10^10^ y) [99], has very low water solubility and strongly adsorb onto organic/inorganic particles, being therefore rather unavailable for biological uptakes [100]. Adsorbed Th enters the human body through inhalation or ingestion or through skin wounds and excessive exposure can induce cancer of the liver, lungs, pancreas, spleen, bone, and blood [101]. Th originates from the air and terrestrial sources to the marine environment [102], and was detected in feedstuffs, such as meat-bone meal and fish meal from aquaculture [103], and in molluscs and fish collected from the Southern Baltic Sea [104,105]. However, none of these studies indicated radiation hazards. Therefore, studies should be conducted aiming at the determination of the Th radionuclides ^228Th^, ^230Th^ and ^232^Th in *S. spallanzanii* and *M. squamiger*, to assess if they pose a radiological hazard.

*Sabella spallanzanii* was proposed as a potential biofilter in the treatment of wastes from intensive aquaculture due to its high capacity to remove particulate organic matter from the water column [106]. In another work, *S. spallanzanii* and the green macroalga *Chaetomorpha linum* (Muller) Kütz. 1849, were cultivated as bioremediators in an integrated multitrophic aquaculture system (IMTA), and obtained biomass was appraised for potential uses [7]. The authors observed a significant increase in the polychaete’s biomass, with high protein levels and adequate fatty acids profile, and suggested that it could partially replace the fishmeal for feeding European seabass (*Dicentrarchus labrax* Linnaeus, 1758) [7]. These results, combined with those obtained in this work, suggest *S. spallanzanii* as a strong candidate to be used in IMTA systems capable to reduce the environmental impact resulting from aquaculture, due to its bioremediation properties, which allow for economic revenues using produced biomass as fish meal ingredient. Similar studies should be conducted targeting *M. squamiger*.

Unravelling lipophilic signatures of *S. spallanzanii* and *M. squamiger* may assist the valorisation of these marine invertebrates as non-traditional sources of functional compounds and fish feed ingredients. Within lipid components, FA as part of molecules or acting individually, have diverse functions in cells that range from structural “building blocks” of cell membranes to suppliers of energy and signalling molecules [107,108].

As far as we know, this is the first report about the FA composition of *M. squamiger*. The overall qualitative FA profiles of *S. spallanzanii* are like those previously described for this polychaete [6,7]. Palmitic acid (C_16:0_) (9) was the major saturated FA found in *S. spallanzanii* and *M. squamiger* methanol and dichloromethane extracts. As previously published studies present relative values of FA, it is not possible to compare the absolute content of compounds and totals of *S. spallanzanii* herein obtained with those of these works. Still, in all previous works, palmitic acid (C_16:0_) (9) was reported as the predominant FA in this species [6,7].

The high content of dihomo-γ-linolenic acid (C_20:3*n*-6c_), EPA (C_20:5*n*-3c_) and DHA (C_22:6*n*-3c_) in dichloromethane extracts of both species open doors for the potential use of these organisms as alternative sources of essential PUFAs for fish feed. The *n*-3 PUFA EPA and DHA are usually included as ingredients in formulations of fish feeds, to ensure adequate fish growth and health, and to improve fish quality for their consumers [109]; however, due to resource depletion, its cost is on the rise [7]. A distinct relative content of *n*-3 and *n*-6 PUFAs were observed in *M. squamiger* and *S. spallanzanii* dichloromethane extracts. The *n*-6 PUFAs relative content found herein for *S. spallanzanii* was higher than those reported in previous studies (around 10%) [7]. An opposite trend was observed in *M. squamiger* dichloromethane extract characterized by a predominance of *n*-3 PUFA over *n*-6 PUFA.

The distinct biological properties attributed to these two families of PUFAs, as for example, cardioprotective action and immunomodulatory effects, make the balance between dietary *n*-6 and *n*-3 fatty acids an important consideration from a human health perspective [109,110]. Indeed, regarding the relationship between *n*-3 and *n*-6 fatty acids, inflammation and disease pathogenesis continue to be subjects of extensive study. While several studies suggest that biological effects of *n*-3 and *n*-6 PUFAs depend on the maintenance of a proper balance between the two series of FA, rather than on the absolute amount of each’s single compound [111], other authors consider that the absolute mass of essential FA consumed, rather than their *n*-6/*n*-3 ratio, should be the main consideration when contemplating lifelong dietary habits affecting health benefits from their intake [109]. In fact, *n*-6 and *n*-3 PUFAs are precursors of potent lipid mediators, called eicosanoids, which play an important role in the regulation of inflammation. Eicosanoids derived from *n*-6 PUFAs (for example, arachidonic acid) have pro-inflammatory and immunoactive functions, whereas eicosanoids derived from *n*-3 PUFAs (for instance, eicosapentaenoic acid (EPA) and docosahexaenoic acid (DHA)) have anti-inflammatory properties, traditionally attributed to their ability to inhibit the formation of *n*-6 PUFA-derived eicosanoids [112]. On the other hand, epidemiological and clinical studies have established that the *n*-6 PUFA linoleic acid (13), and the *n*-3 PUFAs, linolenic acid (21), eicosapentaenoic acid (EPA) (18) and docosahexaenoic acid (DHA) (24), collectively protect against coronary heart diseases [109]. Present Western diets are deficient in *n*-3 fatty acids and have excessive amounts of *n*-6 fatty acids compared with the diet on which human beings evolved and established their genetic patterns. The high *n*-6/*n*-3 PUFA ratio (of around 15-20:1) currently found in today’s Western diets promotes the pathogenesis of many diseases, including cardiovascular disease, inflammatory and autoimmune diseases, and cancer, whereas increased levels of *n*-3 PUFA (a low *n*-6/*n*-3 ratio) exert suppressive effects [113]. Very low *n*-6/*n*-3 ratios were found herein for *M. squamiger* dichloromethane and methanol extracts, as well as for *S. spallanzanii* methanol extract, which agrees with the previous report by Stabili et al. [7]. A higher *n*-6/*n*-3 ratio was found for *S. spallanzanii* dichloromethane extract, yet still below the maximum dietary *n*-6/*n*-3 ratio recommended by the World Health Organization (<10) [114]. The results herein obtained showed the potential of these marine invertebrate specimens for nutritional purposes.

Carotenoids are the most common group of pigments in marine settings, such as those observed in the terrestrial environment [115]. They are synthesized by autotrophic marine organisms, such as bacteria, algae, and fungi, while marine animals can directly accumulate them from food or partly modify them through metabolic mechanisms [115]. Carotenoids are vital for marine animals, since they display key photoprotective and antioxidant functions through the dissipation of light energy, and free radical detoxification, especially in shallow habitats characterized by sporadic exposure to excessive solar radiation [116,117]. Carotenoids are also important in animals’ nutrition due to their roles in provitamin A activity and immunological modulation [117]. No carotenoids were detected in *S. spallanzanii*, while fucoxanthinol (1) and fucoxanthin (2) were the major compounds in the extracts from *M. squamiger*. Fucoxanthin is an abundant marine carotenoid in edible brown algae, such as *Undaria pinnatifida*, *Hijikia fusiformis*, *Saccharina japonica*, and *Sargassum horneri* [118,119], and was also identified in marine invertebrates, as for example the ascidian *Botryllus schlosseri* (Pallas, 1766) from the Black Sea [120]. Fucoxanthin is considered as one of the most potent carotenoids regarding anti proliferative activity in human cancer cells and exhibited no toxicity in rodent models [121]. Other beneficial health effects of fucoxanthin include regulation of obesity, diabetes, and inflammation [87]. Fucoxanthinol is a major gastrointestinal metabolite of dietary fucoxanthin, and to date, the number of natural sources of this compound is reduced, including, for example, the diatom *Nitzschia laevis* [122,123]. Fucoxanthinol also plays essential roles in the regulation of obesity, and displays relevant health promoting properties, such as antidiabetic, anti-tumoral and anti-inflammatory [124].

Carotenoids have a high demand in the cosmetics, pharmaceutical and food areas, due to their multiple health enhancement properties. In fact, estimated value for the global carotenoid market in 2016 was approximately 1.20 billion USD, which is expected to reach 1.53 billion USD by 2021, at a compound annual growth rate (CAGR) of 3.78% from 2016 to 2021 [125]. They are also often used in aquaculture to reinforce fish colour, which increases the consumer’s perception of good quality and organoleptic properties; furthermore, they are considered semi-essential nutrients that promote optimal fish survival and growth at relatively low dietary-inclusion levels, due to their several functions in fish, such as antioxidant, anti-inflammatory, anti-stress, and immunomodulation agents [126]. Therefore, the carotenoid contents of *M. squamiger* could be a high added asset if considering the use of this species as a source of bioactive fish meal ingredient.

In this work, *S. spallanzanii* and *M. squamiger* were evaluated for the first time for antioxidant properties, by *in vitro* methods, targeting free radicals and redox metals, namely Cu and Fe. The methanol extract of *S. spallanzanii* exhibited metal chelating properties towards both metals, while the methanol extract from *M. squamiger* was able to chelate Cu ions. Iron and Cu are essential elements for fish metabolism; however, their excess can induce oxidative stress in aquatic organisms by promoting the formation of reactive oxygen species (ROS) that may cause oxidative damage to proteins and lipids, which is deleterious to the overall fish health, fish quality and suitability for human consumption [127,128]. Metal chelators, such as EDTA, are used in aquaculture to counteract metal accumulation. For example, dietary EDTA supplementation in Nile tilapia (*O. niloticus*) was used and its benefits were attributed to the enhancement of the antioxidant activity and reduction of heavy metal bioaccumulation in fish [129].

All samples showed antioxidant activity in the ORAC assay, and the obtained values were lower than those obtained with extracts from other marine invertebrates, such as the Atlantic sea cucumber (*Cucumaria frondosa* (Gunnerus 1767) (values ranging between 35 and 200 mg TE/g) [130]. In aquaculture, oxidative stress leads to damages in biomolecules, causing immune suppression, pathological symptoms, and slow growth in fish; it results from several factors, such as high temperature and hypoxia [131]. Synthetic antioxidants such as ethoxyquin have been widely included in fish feed to counteract oxidative stress and promote fish health, and to improve the feed quality and shelf life [132]. However, the uncertainty of the health implication of synthetic antioxidants implies the need for efficient and cost effective alternative natural sources [132]. Our results suggests that *S. spallanzanii* and *M. squamiger* contain polar molecules with metal chelation and antioxidant properties, and, therefore, could be explored as sources of ingredients in fishmeal, as natural metal chelators and to counteract oxidative stress. These results, combined with those previously reported in this work, suggest both species as sources of ingredients with high added value to be used for different commercial purposes, and raise the interest in using biomass from both species collected from the wild, to control its populations, or to produce them by sustainable methods. As stated previously, *S. spallanzanii* can be successfully produced by IMTA [7]; however, there are no reports on the production of *M. squamiger*. There are, however, reports of the breeding of other ascidians, such as the tunicate *Botrylloides diegensis* Ritter & Forsyth, 1917, in recirculating artificial seawater conditions [133].

## 5. Conclusions

Our results indicate that *S. spallanzanii* contains appreciable levels of ash and protein, a predominance of the amino acids arginine and glycine, of the minerals Na, Ca, and K, and of the FA dihomo-γ-linolenic, palmitoleic and palmitic acids The methanol extract of *S. spallanzanii* exhibited Cu and Fe chelating properties, and capacity to scavenge peroxyl free radicals. *Microcosmus squamiger* had high total protein levels, was enriched in the amino acid taurine, in the minerals Na, Ca and K, and in the FA eicosapentaenoic, docosahexaenoic, elaidic and palmitic acids. Fucoxanthinol and fucoxanthin were the major carotenoids in the *M. squamiger* dichloromethane extract. The methanol extract from *M. squamiger* exhibited Cu chelating properties, while both extracts were able to scavenge peroxyl free radicals. Overall, our results bring new insights into the possible use of *S. spallanzanii* and *M. squamiger* as sources of bioactive ingredients with different potential commercial applications, such as in the biomedical, food and feed areas. For example, *S. spallanzanii* biomass could be incorporated in fish feed as an attractant, while *M. squamiger* could be used as a taurine supplementation, in aquaculture.

## Figures and Tables

**Table 1:** Proximate composition and energetic value of the biomass of *Sabella spallanzanii* and *Microcosmus squamiger*.

Item	*S. spallanzanii*	*M. squamiger*
Moisture (%)	70.7 ± 1.0	88.9 ± 0.8
Ash (%)	53.1 ± 4.8	36.2 ± 4.6
Protein (%, dw)	47.2 ± 0.3	53.9 ± 2.6
Fat (%, dw)	11.7 ± 1.7	5.3 ± 0.1
Carbohydrates (%, dw)	41.1 ± 1.4	40.8 ± 2.6
Energetic value (kcal/100 g, dw)	475.8 ± 8.1	445.6 ± 0.4

Data represent the mean ± standard error of mean (SEM) (*n* = 3). dw: dry weight.

**Table 2:** Amino acid profile of the biomass of *Sabella spallanzanii* and *Microcosmus squamiger*.

Amino Acid	Abbreviation	*S. spallanzanii*	*M. squamiger*
Arginine	Arg	68.5 ± 0.33	1.65 ± 0.01
Histidine	His	0.27 ± 0.00	0.75 ± 0.01
Lysine	Lys	0.25 ± 0.00	1.29 ± 0.02
Threonine	Thr	0.69 ± 0.00	1.31 ± 0.02
Isoleucine	Ile	0.12 ± 0.00	0.17 ± 0.00
Leucine	Leu	0.20 ± 0.00	0.27 ± 0.00
Valine	Val	0.21 ± 0.01	0.73 ± 0.00
Tryptophan	Trp	0.03 ± 0.00	0.23 ± 0.00
Methionine	Met	0.19 ± 0.00	0.85 ± 0.01
Phenylalanine	Phe	0.19 ± 0.00	0.25 ± 0.00
Cysteine	Cys	0.08 ± 0.00	0.15 ± 0.00
Tyrosine	Tyr	0.14 ± 0.00	0.33 ± 0.00
Aspartic acid	Asp	1.39 ± 0.01	0.92 ± 0.03
Asparagine	Asn	0.61 ± 0.01	0.67 ± 0.01
Glutamic acid	Glu	2.02 ± 0.02	2.41 ± 0.05
Glutamine	Gln	1.71 ± 0.01	0.88 ± 0.00
Alanine	Ala	1.06 ± 0.00	2.94 ± 0.03
Glycine	Gly	49.3 ± 0.16	1.72 ± 0.03
Proline	Pro	1.14 ± 0.03	5.15 ± 0.06
Serine	Ser	0.62 ± 0.00	0.82 ± 0.01
Taurine	Tau	2.30 ± 0.07	26.6 ± 0.46
Ornithine	Orn	0.19 ± 0.00	0.65 ± 0.01
gamma-Amino-n-butyric acid	GABA	0.13 ± 0.00	0.45 ± 0.01
Hydroxyproline	HPro	0.18 ± 0.00	0.95 ± 0.00
beta-Alanine	B-Ala	0.14 ± 0.00	0.23 ± 0.00
TOTAL		131.76 ± 0.66	52.42 ± 0.78

Data represent the mean ± standard error of mean (SEM) (*n* = 2) and is expressed as mg/g dry weight (dw).

**Table 3:** Mineral content of *Sabella spallanzanii* and *Microcosmus squamiger*.

Mineral	Symbol	*S. spallanzanii*	*M. squamiger*
Macroelements			
Sodium	Na	18.65 ± 0.26	36.24 ± 0.64
Calcium	Ca	17.09 ± 0.38	14.29 ± 1.11
Potassium	K	15.14 ± 0.10	11.88 ± 0.36
Magnesium	Mg	6.66 ± 0.05	6.75 ± 0.14
Phosphorus	P	3.97 ± 0.01	2.66 ± 0.15
Microelements			
Iron	Fe	12.12 ± 0.17	4.42 ± 0.30
Selenium	Se	nd	nd
Manganese	Mn	nd	0.41 ± 0.02
Molybdenum	Mo	nd	nd
Aluminum	Al	13.10 ± 0.27	4.60 ± 0.17
Lithium	Li	nd	nd
Vanadium	V	11.6 ± 0.43	4.63 ± 0.17
Toxic elements			
Chromium	Cr	nd	nd
Copper	Cu	nd.	nd
Zinc	Zn	nd	nd
Cadmium	Cd	nd	nd
Nickel	Ni	nd	nd
Lead	Pb	nd	nd
Mercury	Hg	nd	nd
Thorium	Th	0.28 ± 0.07	0.48 ± 0.05
TOTAL		98.61	86.36

Data represents the mean ± standard error of mean (SEM) (*n* = 3) and values are expressed as mg/g dry weight (dw). nd: not detected.

**Table 4:** Fatty acid content of *Sabella spallanzanii* and *Microcosmus squamiger* extracts (µg/mg dry extract, dw) ^1^.

			*S. spallanzanii*				*M. squamiger*			
Compound	Common Name	Peak	Dichloromethane Extract		Methanol Extract		Dichloromethane Extract		Methanol Extract	
			µg/mg, dw	Relative%	µg/mg, dw	Relative%	µg/mg, dw	Relative%	µg/mg, dw	Relative%
SFA										
C_11:0_	Undecylic acid	1	–	–	0.06 ± 0.00	0.3	–	–	–	–
C_12:0_	Lauric acid	2	nq	nq	nq	nq	–	–	nq	nq
C_13:0_	Tridecyclic acid	3	–	–	nq	nq	–	–	nq	nq
C_14:0_	Myristic acid	5	33.71 ± 4.10	7.3	3.11 ± 0.14	17.4	8.61 ± 0.22	3.3	1.00 ± 0.04	5.3
C_15:0_	Pentadecyclic acid	7	5.36 ± 0.09	1.2	0.56 ± 0.05	2.6	2.75 ± 0.19	1.0	0.49 ± 0.02	2.6
C_16:0_	Palmitic acid	9	89.35 ± 7.13	19.5	5.21 ± 0.13	24.2	43.55 ± 1.57	16.5	6.32 ± 0.81	33.1
C_17:0_	Margaric acid	11	3.87 ± 0.26	0.8	0.52 ± 0.07	2.4	5.47 ± 0.83	2.1	0.83 ± 0.11	4.3
C_18:0_	Stearic acid	16	18.54 ± 3.25	4.0	2.18 ± 0.18	10.1	15.63 ± 1.31	5.9	3.02 ± 0.35	15.8
C_20:0_	Arachidic acid	22	0.75 ± 0.08	0.2	0.43 ± 0.09	2.0	0.40 ± 0.02	0.2	0.34 ± 0.07	1.8
C_21:0_	Heneicosylic acid	23	nq	–	0.40 ± 0.01	1.8	nq	nq	0.74 ± 0.07	3.9
C_22:0_	Behenic acid	27	0.33 ± 0.10	0.1	nq	nq	nq	nq	nq	nq
C_23:0_	Tricosylic acid	28	nq	nq	nq	nq	nq	nq	0.02 ± 0.00	0.1
C_24:0_	Lignoceric acid	30	–	–	nq	nq	nq	nq	0.02 ± 0.01	0.1
Σ SFA			151.91 ± 15.01	33.1	12.47 ± 1.60	57.8	76.41 ± 4.13	30.4	12.78 ± 1.48	**66.9**
MUFA										
C_14:1*n*-5c_	Myristoleic acid	4	nq	nq	–	–	–	–	nq	nq
C_15:1*n*-5c_	cis-10-Pentadecenoic acid	6	–	–	–	–	–	–	nq	nq
C_16:1*n*-7c_	Palmitoleic acid	8	44.64 ± 3.10	9.7	1.52 ± 0.12	7.0	20.46 ± 2.99	3.0	0.97 ± 0.09	5.1
C_17:1*n*-7c_	cis-10-Heptadecenoic acid	10	–	–	–	–	–	–	nq	nq
C_18:1*n*-9c_	Oleic acid	14	20.42 ± 1.25	4.4	0.33 ± 0.06	1.5	9.20 ± 0.30	3.5	0.72 ± 0.06	3.8
C_18:1*n*-9t_	Elaidic acid	15	32.34 ± 1.25	7.0	1.68 ± 0.13	7.8	25.65 ± 3.73	9.7	2.56 ± 0.28	13.4
C_22:1*n*-9c_	Erucic acid	26	0.48 ± 0.06	0.1	nq	nq	nq	nq	nq	nq
C_24:1*n*-9c_	Nervonic acid	29	0.43 ± 0.09	0.1	–	–	–	–	–	–
Σ MUFA			98.31 ± 5.75	21.4	3.53 ± 0.39	16.4	55.31 ± 7.01	22.0	4.25 ± 0.43	**22.3**
PUFA										
C_18:2*n*-6c_	Linoleic acid	13	4.35 ± 0.08	0.9	0.04 ± 0.02	0.2	8.39 ± 0.34	3.2	0.68 ± 0.05	3.5
C_18:2*n*-6t_	Linoelaidic acid	25	2.42 ± 0.35	0.5	0.06 ± 0.01	0.3	0.24 ± 0.02	0.1	nq	0
C_20:2*n*-6c_	Eicosadienoic acid	20	23.27 ± 3.44	5.1	0.94 ± 0.07	4.4	0.32 ± 0.08	0.1	0.06 ± 0.00	0.3
C_18:3*n*-6c_	γ-Linolenic acid	12	nq	nq	nq	nq	nq	nq	nq	nq
C_18:3*n*-3c_	α-Linolenic acid	21	10.08 ± 0.86	2.2	0.44 ± 0.04	2.0	0.46 ± 0.10	0.2	0.13 ± 0.02	0.7
C_20:3*n*-6c_	Homo-γ-Linolenic acid	19	124.69 ± 11.8	27.2	nq	nq	nq	nq	nq	nq
C_20:4*n*-6c_	Arachidonic acid	17	21.24 ± 2.69	4.6	1.17 ± 0.02	5.4	11.20 ± 0.93	4.2	0.39 ± 0.04	2.1
C_20:5*n*-3c_	Eicosapentaenoic acid	18	*	*	2.39 ± 0.34	11.1	59.11 ± 8.87	22.4	0.56 ± 0.05	2.9
C_22:6*n*-3c_	Docosahexaenoic acid	24	22.97 ± 3.23	5.0	0.51 ± 0.04	2.4	39.92 ± 6.67	15.1	0.23 ± 0.02	1.2
Σ PUFA			209.03 ± 22.45	45.5	5.56 ± 0.69	25.8	119.64 ± 17.01	47.6	2.05 ± 0.18	10.7
Σ *n*-3			33.05 ± 4.09	7.2	3.34 ± 0.58	15.5	99.48 ± 5.64	39.6	0.92 ± 0.09	4.8
Σ *n*-5			nq	nq	nd	nd	nd	nd	nq	nq
Σ *n*-6			175.98 ± 18.36	38.2	2.22 ± 0.11	10.3	20.16 ± 1.37	8.0	1.13 ± 0.09	5.9
Σ *n*-7			44.64 ± 3.1	9.7	1.52 ± 0.12	7.0	20.46 ± 2.99	8.1	0.97 ± 0.09	5.1
Σ *n*-9			53.67	11.7	2.01 ± 0.27	9.3	34.85 ± 4.02	13.9	3.29 ± 0.34	17.2
*n*-6/*n*-3			5.33		0.67		0.20		1.23	
TOTAL			459.25 ± 43.20	100.0	21.56 ± 2.68	100.0	251.34 ± 28.15	100.0	19.09 ± 2.09	100.0

^1^ Results are expressed as the mean ± standard deviation (SD) (*n* = 3), as µg/mg dry weight (dw). SFA: saturated fatty acid; MUFA: monounsaturated fatty acid; PUFA: polyunsaturated fatty acid; nq: not quantified; “–“: not detected; *∑* sum. * Quantified with C_20:3*n*-6c_ (19).

**Table 5:** Carotenoid content of *Sabella spallanzanii* and *Microcosmus squamiger* extracts (µg/g dry extract) ^1^.

Peak	Compound	*M. squamiger*		*S. spallanzanii*	
		Dicloromethane Extract	Methanol Extract	Dicloromethane Extract	Methanol Extract
1	Fucoxanthinol	2.51 ± 0.28	nd	nd	nd
2	Fucoxanthin	1.66 ± 0.06	nd	nd	nd
3	Anteraxanthin	nq	nd	nd	nd
4	Astaxanthin	0.03 ± 0.00	nd	nd	nd
5	Lutein	0.14 ± 0.01	nd	nd	nd
6	Zeaxanthin	nq	nd	nd	nd
	TOTAL	4.34 ± 0.35	–	–	–

^1^ Results are expressed as the mean ± standard deviation (SD) (*n* = 3), as µg/g dry weight (dw). nq: not quantified; nd: not detected. Peaks refers to the HPLC-DAD profile depicted in Figure 3.

**Table 6:** Extraction yields (%), DPPH radical scavenging activity and metal chelating activities on iron (ICA) and copper (CCA) (IC_50_, mg/mL) of dichloromethane and methanol extracts of *Sabella spallanzanii* and *Microcosmus squamiger*.

Samples	Extract	Yield (%)	DPPH	ICA	CCA
*S. spallanzanii*	Dichloromethane	5.06	nr	nr	nr
	Methanol	6.32	nr	4.1 ± 0.1 ^b^	1.7 ± 0.0 ^c^
*M. squamiger*	Dichloromethane	2.42	nr	nr	>1 nr
	Methanol	15.65	nr	nr	7.6 ± 0.2 ^b^
BHA *	-	-	0.1 ± 0.0	-	-
EDTA *	-	-	-	0.1 ± 0.0 ^a^	0.6 ± 0.0 ^a^

Data represents the mean ± standard error of mean (SEM) (*n* = 6). In the same column, values followed by different letters are significantly different at *p* < 0.05 (one-way ANOVA with Tukey post hoc test); nr: IC_50_ value not reached; * positive control; BHA: butylated hydroxyanisole; EDTA: ethylenediaminetetraacetic acid.

## Data Availability

The dataset is available upon request from the corresponding author.

## Supplementary Materials

The following are available online at https://www.mdpi.com/article/10.3390/ani11123557/s1, Table S1: Linearity, limit of detection (LOD) and limit of quantification (LOQ) of FAME with the employed analytical conditions for carotenoids determination; Table S2 Regression equation, linearity, limit of detection (LOD) and limit of quantification (LOQ) of reference compounds with the employed analytical conditions for the carotenoid determination.
