# Supplementary material for: Exploring the Biotechnological Value of Marine Invertebrates: A Closer Look at the Biochemical and Antioxidant Properties of Sabella spallanzanii and Microcosmus squamiger
Source: Animals (Basel). 2021 Dec 14;11(12):3557. doi: 10.3390/ani11123557 (PMC8697903; doi:10.3390/ani11123557)
Supplement: Supplementary file 1 [file animals-11-03557-s001.zip › animals-1438064-supplementary.pdf]

Table S1. Linearity, limit of detection (LOD) and limit of quantification (LOQ) of FAME with the employed analytical conditions for carotenoids determination.

Compound [a]	Regression equation	r^2	Linearity (µg/mL)	LOD (µg/mL)	LOQ (µg/mL)
$C_{11:0}$	$y = 1.08x + 3.35$	0.996	1.35 – 86.50	0.30	0.91
$C_{12:0}$	$y = 0.91x + 8.34$	0.994	3.16 – 202.50	1.18	3.57
$C_{13:0}$	$y = 0.89x + 4.72$	0.993	1.59 – 101.50	0.50	1.52
$C_{14:0}$	$y = 0.87x + 9.94$	0.992	3.16 – 202.00	0.66	2.01
$C_{14:1n\text{-}5c}$	$y = 0.86x + 4.89$	0.992	1.58 – 101.00	0.33	1.01
$C_{15:0}$	$y = 0.83x + 5.23$	0.989	1.58 – 101.00	0.11	0.35
$C_{15:1n\text{-}5c}$	$y = 0.83x + 5.01$	0.990	1.59 – 102.00	0.09	2.01
$C_{16:0}$	$y = 0.82x + 15.67$	0.990	4.78 – 306.00	0.64	1.93
$C_{16:1n\text{-}7c}$	$y = 0.80x + 5.22$	0.990	1.59 – 102.00	0.12	0.35
$C_{17:0}$	$y = 0.78x + 2.20$	0.992	0.79 – 50.50	0.29	0.89
$C_{17:1n\text{-}7c}$	$y = 0.82x + 4.83$	0.991	1.59 – 102.00	0.43	1.30
$C_{18:0}$	$y = 0.81x + 8.31$	0.992	3.19 – 204.00	1.75	5.29
$C_{18:1n\text{-}9c}$	$y = 1.67x + 17.28$	0.992	3.18 – 203.50	1.37	4.14
$C_{18:1n\text{-}9t}$	$y = 0.77x + 4.55$	0.993	1.58 – 101.00	0.80	2.43
$C_{18:2n\text{-}6c}$	$y = 0.85x + 4.17$	0.993	1.59 – 101.50	0.59	1.78
$C_{18:2n\text{-}6t}$	$y = 0.89x + 1.88$	0.989	1.58 – 101.00	0.56	1.71
$C_{18:3n\text{-}6c}$	$y = 0.83x + 4.13$	0.993	0.73 – 2.21	1.58	101.00
$C_{18:3n\text{-}3c}$	$y = 1.58x + 6.36$	0.990	1.58 – 101.00	0.76	2.31
$C_{20:0}$	$y = 0.82x + 6.59$	0.990	3.19 – 204.00	1.64	4.98
$C_{20:2n\text{-}6c}$	$y = 0.81x + 2.67$	0.994	1.56 – 100.00	0.67	2.21
$C_{20:3n\text{-}6c}$	$y = 1.00x + 3.20$	0.992	1.27 – 81.50	0.64	1.94
$C_{20:4n\text{-}6c}$	$y = 0.70x + 2.67$	0.993	1.58 – 101.00	0.95	2.87
$C_{20:5n\text{-}3c}$	$y = 1.07x + 2.87$	0.993	1.09 – 70.00	0.50	1.51
$C_{21:0}$	$y = 0.84x + 2.30$	0.990	1.59 – 101.50	0.65	1.97
$C_{22:0}$	$y = 0.85x + 3.65$	0.989	3.16 – 202.50	1.15	3.47
$C_{22:1n\text{-}9c}$	$y = 0.81x + 1.92$	0.988	1.59 – 101.50	0.63	1.91
$C_{22:6n\text{-}3c}$	$y = 1.02x + 1.84$	0.992	1.05 – 67.00	0.38	1.16
$C_{23:0}$	$y = 1.08x + 1.14$	0.988	1.29 – 82.50	0.35	1.05
$C_{24:0}$	$y = 0.90x + 1.07$	0.989	3.16 – 202.50	0.83	2.51
$C_{24:1n\text{-}9c}$	$y = 0.90x + 0.50$	0.988	1.59 – 102.00	0.47	1.42

[a] Parent fatty acid.

Table S2. Regression equation, linearity, limit of detection (LOD) and limit of quantification (LOQ) of reference compounds with the employed analytical conditions for the carotenoid determination.

Peak	Compound	Regression Equation (µg/mL)	r^2	Linearity (µg/mL)	LOD (µg/mL)	LOQ (µg/mL)
1	Fucoxanthinol	$y = 2.37{\times}10^5\ x + 720053\ 16$	1.000	4.12 – 333.00	0.77	2.35
2	Fucoxanthin	$y = 5.86{\times}10^5\ x + 683281\ 56$	1.000	111.11 – 1.37	0.29	0.88
3	Anteraxanthin	$y = 3.56{\times}10^6\ x + 327133\ 16$	0.997	0.20 – 3.13	0.01	0.02
4	Astaxanthin	$y = 2.52{\times}10^6\ x + 459278,\ 4$	0.999	0.81 – 13.00	0.37	1.12
5	Lutein	$y = 1.55{\times}10^6 x – 130964\ 47$	1.000	0.45 – 36.33	0.09	0.29
6	Zeaxanthin	$y = 2.94{\times}10^{96}\ x + 372456,\ 2$	0.999	0.20 – 3.13	0.01	0.02

LOD – limit of detection. LOQ – limit of quantification.
